# Thyroid Paraganglioma With Medullary Carcinoma: A Unique Combination in a Patient in Association With Multiple Endocrine Neoplasia Type 2B Syndrome With Prolonged Survival

**DOI:** 10.7759/cureus.28423

**Published:** 2022-08-26

**Authors:** Eleni Thodou, Theodossia Choreftaki, Theodora Kounadi, Labrini Papanastasiou, George Kontogeorgos

**Affiliations:** 1 Department of Pathology/Cytology, University of Thessaly, School of Medicine, Larissa, GRC; 2 Department of Pathology, “G. Gennimatas” Athens General Hospital, Athens, GRC; 3 Department of Endocrinology, “G. Gennimatas” Athens General Hospital, Athens, GRC; 4 First Propaepeudic Department of Internal Medicine, Laikon Hospital, National and Kapodistrian University of Athens School of Medicine, Athens, GRC

**Keywords:** metastases, prolonged survival, ret mutations, tyrosine kinase inhibitors (tki), c-cell hyperplasia, genetics, men 2b, medullary carcinoma, paraganglioma, thyroid

## Abstract

Head and neck paragangliomas (PGLs) most commonly derive from the carotid body, jugulotympanic, vagal, and laryngeal paraganglia. Thyroid PGLs originate in the inferior laryngeal paraganglion, which may lie inside the thyroid parenchyma. Intrathyroid PGLs are rare with approximately 75 cases reported to date, mostly as solitary lesions. The coexistence of thyroid PGL with medullary thyroid carcinoma (MTC) has not been reported. Here, we report a unique case of intrathyroid PGL concomitant with MTC in the context of multiple endocrine neoplasia type 2B syndrome. Interestingly, the patient showed a prolonged survival with good clinical response to tyrosine kinase inhibitors, despite her advanced metastatic MTC. We discuss the challenges in pathology, differential diagnosis, and genetic background for the development of these thyroid lesions.

## Introduction

Paraganglioma (PGL) is a non-epithelial neuroendocrine tumor originating from the paraganglia of the sympathetic or parasympathetic autonomous nervous system. Head and neck PGLs derive from the carotid body, jugulotympanic, vagal, and laryngeal paraganglia. Thyroid PGLs develop from the inferior laryngeal paraganglion, which may lie inside the thyroid parenchyma, or be retracted toward the thyroid gland by the recurrent laryngeal nerve [1,2]. Intrathyroid PGL is a rare entity, with approximately 75 cases being reported, the majority being solitary lesions [3,4]. The coexistence of thyroid PGL with thyroid carcinoma is extremely rare, and a single case of papillary carcinoma has been reported to date [5].

Here, we describe a case of primary thyroid PGL concomitant with medullary thyroid carcinoma (MTC) in a young patient with multiple endocrine neoplasia type 2 (MEN 2B) syndrome documented by molecular testing. To our knowledge, this combination has never been described before in MEN 2B. We discuss the pathology, differential diagnosis, and genetic background of these lesions and review the literature.

## Case presentation

A 16-year-old female was admitted to the Department of Gastroenterology of “G. Gennimatas” Athens General Hospital for investigation of long-standing chronic diarrhea and suspected Crohn’s disease. Endoscopic investigation revealed megacolon, and subsequent biopsies confirmed Crohn’s disease. During her hospitalization, she developed respiratory failure with pleural and pericardial effusion. A cervical and thoracic computerized tomography (CT) scan revealed an enlarged thyroid gland and enlarged cervical and mediastinal lymph nodes.

The patient was then referred to the Department of Endocrinology for further investigation. A thyroid ultrasound (US) examination revealed nodules on both thyroid lobes; a hypoechoic nodule measuring approximately 3.5 cm in maximal diameter on the left lobe, and a well-defined hypoechoic nodule measuring 1.3 cm on the right lobe. Cytology of fine-needle aspiration biopsy performed only on the larger nodule reported malignancy consistent with an MTC. Serum calcitonin and carcinoembryonic antigen (CEA) levels were also very high (1,330 pg/mL and 332 ng/mL, respectively). A 24-hour urine catecholamine measurement was normal.

**Figure 1:**
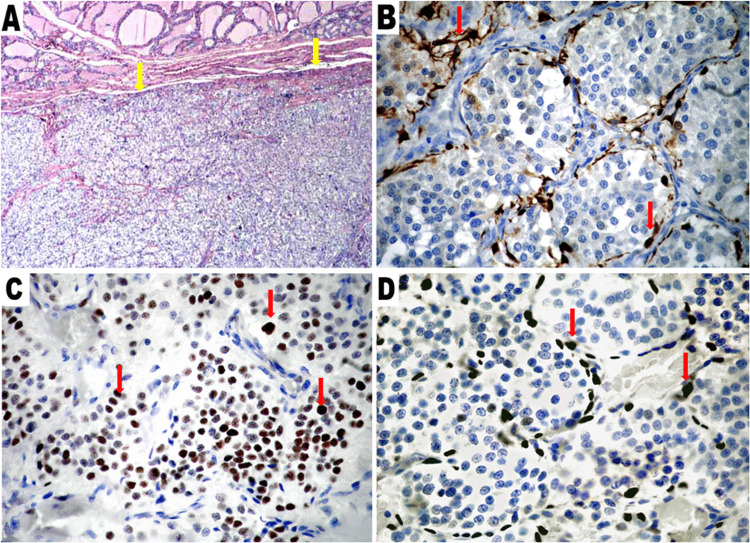
Thyroid paraganglioma. A: Aspect of PRG/thyroid parenchyma interface. The two components are separated by fibrous band (H&E, 2.5×). B: S-100 protein depicts sustentacular cells surrounding round-shaped cell nests with characteristic “Zellballen” pattern. The sustentacular cells display attenuated anastomosing cellular processes (20×). C: PGL cells with selective nuclear immunoreactivity for GATA3 (20×). D: Sustentacular cells with strong nuclear immunoreactivity for SOX10 (20×). PGL: paraganglioma; GATA3: GATA binding protein 3; SOX10: SRY-related HMG-box 10

Clinically, she showed Marfanoid features and multiple oral mucosal neuromas, particularly on the lips and tongue. Total thyroidectomy with extensive lymph node dissection was performed. On sectioning, the smaller nodule on the upper pole of the right lobe was circumscribed whitish. On histology, the nodule in the right lobe showed morphological features of PGL with the typical “Zellballen” pattern. Immunostaining was positive for synaptophysin, chromogranin A, and GATA binding protein 3 (GATA3) and negative for cytokeratins and calcitonin. S-100 protein and SRY-related HMG-box 10 (SOX10) immunostains selectively depicted sustentacular cells surrounding the neoplastic cell nests (Figure 1). The Ki-67 proliferation index was estimated at 2%.

**Figure 2:**
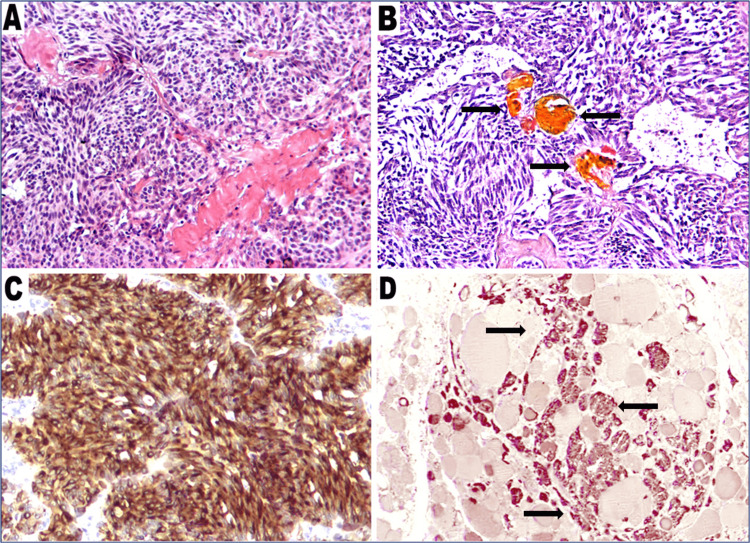
Medullary carcinoma. A: MTC, mostly composed of spindle cells forming closely packed solid nests. Amorphous eosinophilic deposits are noted in the stroma (H&E, 10×). B: Massive amyloid deposits showing green-yellowish birefringence under polarized light (Congo red, 10×). C: Closely packed solid nests with strong immunopositivity for calcitonin (10×). D: Multiple foci of C-cell hyperplasia in the thyroid parenchyma immunoreactive for calcitonin (2.5×). MTC: medullary thyroid carcinoma; H&E: hematoxylin and eosin

The neoplasm on the left lobe was solid, whitish to brownish with ill-defined borders. Histology confirmed an MTC composed of mostly spindle and partly round cells. Focal depositions of amorphous eosinophilic material were noted in the stroma, which were positive on Congo red. Multiple solid nests of C-cell hyperplasia were noted in the adjacent parenchyma. Extensive metastases were observed in the majority of the lymph nodes excised. On immunohistochemistry, the tumor cells were positive for calcitonin and keratin 8, while thyroglobulin and S-100 protein were negative. In addition, immunostain for calcitonin was positive in the foci of C-cell hyperplasia (Figure 2).

The metastatic MTC with very early onset with synchronous thyroid PGL in combination with oral neuromas and a Marfanoid body habitus were strongly indicative of MEN syndrome. Polymerase chain reaction-based genetic analysis of the DNA extracted from blood lymphocytes, using the specific restriction enzyme Fok1, disclosed a methionine-to-threonine substitution at codon 918 on exon 16 of RET protooncogene (M918T RET) heterozygous mutation, which is diagnostic of MEN 2B syndrome.

During the next four years, she underwent three consecutive debulking surgeries with resection of cervical and mediastinum lymph nodes for persistent metastatic disease with very high calcitonin and CEA serum levels. The patient consequently developed multiple hepatic, lung, and osseous metastasis (stage IVc). Six years after the initial diagnosis and after the introduction of tyrosine kinase inhibitors (TKI) as therapeutic modalities for metastatic MTC, she was started on therapy with cabozantinib (140 mg/day). A year later, the patient experienced colon perforation as a side effect and underwent partial colectomy. The therapy was altered to vandetanib 300 mg/day initially, but later, due to gastrointestinal side effects, it was reduced to 200 mg/day. Metastatic foci diminished dramatically, and calcitonin and CEA levels declined from 58,000 to 6,000 pg/mL and from 747 μU/L to 200 μU/L, respectively. The patient has been alive for 19 years after the initial diagnosis with stable metastatic disease.

## Discussion

Thyroid PGLs account for approximately 0.5% of all head and neck PGLs. They typically occur in patients with a mean age of 40-50 years, with a male-to-female ratio of 1:8. They mostly present as single, slowly growing nodules, showing hypoechogenicity and hypervascularity on US [4,6]. They are usually benign, encapsulated neoplasms measuring 2-10 cm, rarely presenting as a rapidly enlarging mass causing local compressive symptoms. Tumors invading the cricoid cartilage, trachea, or esophagus, as well as malignant tumors with metastases, are infrequently reported. As a rule, they are non-functioning deriving from the parasympathetic paraganglia [6]. Symptoms of catecholamine hypersecretion, as hypertension, tachycardia, and weight loss are exceptional [7].

Primary thyroid PGLs are located within the thyroid parenchyma. For establishing the diagnosis, the possibility of a tumor of the carotid body or other cervical paraganglia compressing the adjacent thyroid parenchyma should be excluded.

The differential diagnosis includes nesting-pattern-forming neoplasms, such as MTC, hyalinizing trabecular tumor, and metastases of neuroendocrine carcinomas to thyroid [1]. In particular, the PGL-like variant of MTC, rarely reported in the literature, mimics the typical “Zellballen” pattern of PGL and may also show sustentacular cells surrounding the cell nests [8,9]. The diagnosis of MTC is established by positive immunostains for calcitonin, CEA, and cytokeratins. In clinical terms, elevated serum calcitonin further supports the diagnosis [8,9]. In our patient, the MTC consisting mostly of spindle cells was distinct from PRG. However, it should be stressed that in cases of multifocal MTC, occasionally encountered in MEN 2 patients, a coexisting thyroid PGL may be overlooked.

In PGLs, general neuroendocrine markers such as chromogranin A and synaptophysin are positive, whereas cytokeratins are usually negative [4,6,10]. Immunohistochemistry positive for GATA3 and negative for cytokeratins is diagnostic for PGL, excluding the possibility of metastatic neuroendocrine carcinoma from other sites [10]. The differential diagnosis of hyalinizing trabecular tumors from PGLs is based on the nuclear features of papillary carcinoma, such as nuclear grooves and pseudoinclusions. On immunohistochemistry, the hyalinizing trabecular tumor is positive for thyroid transcription factor 1 and thyroglobulin and negative for neuroendocrine markers [1].

In approximately 30-40% of cases, PGLs are associated with a germline genetic disorder, mostly familial PGL 1 to PGL 5 syndromes, caused by the mutations of genes encoding for succinate dehydrogenase (SDH) [11]. Regarding thyroid PGLs, a genetic predisposition has been documented in 80% of the cases, related to *SDHA *and *SDHB *mutations [4]. Head and neck PGLs are also encountered in syndromes associated with multiple tumorigenesis, such as von Hippel-Lindau, MEN 2, and neuroﬁbromatosis type 1, caused by germline or somatic mutations in *VHL*, *RET*, and *NF1* genes, respectively [12].

Our patient presented with signs of MEN 2B syndrome and had no family history of any genetic disorder. MEN 2B is rare, invariably associated with MTC, while about 50% of the patients develop pheochromocytomas (PCCs) [13].

*RET* mutations are responsible for MEN 2B syndrome with *M918T RET* accounting for more than 95% of the cases. In most MEN 2B patients, *RET *mutations develop de novo [14]. MTC in patients carrying the *M918T RET* mutation occurs earlier in life and is more aggressive. In addition, these patients also present with one or more extra endocrine features, such as Marfanoid body habitus, skeletal anomalies, multiple neuromas of the oral cavity, thickened corneal nerves, and chronic gastrointestinal symptoms from early childhood [13]. Early diagnosis of MEN 2B in childhood remains a challenge due to the unfamiliarity of this rare syndrome. Given the lack of family history in the majority of cases, the diagnosis relies on clinical grounds alone [15].

Further, other reasons for delayed diagnosis are lack of endocrine signs in early childhood and gradual development of the characteristic clinical manifestations with age. If at least one of the clinical stigmata are recognized in young age, calcitonin levels should be measured together with a genetic test for *RET* mutations [13,16]. Although MTC in this syndrome has an early onset, even in the first year of life, it is typically diagnosed in the second decade with a mean age of 16 years [14,15]. In concordance with the literature, our patient had a history of long-standing diarrhoeic symptoms since the age of 10 and was diagnosed with Crohn’s disease by the time MTC was also detected at the age of 16. She also showed typical non-endocrine clinical features of MEN 2B, which was confirmed by the genetic profile of *M918T RET* mutation. The exceptional finding in our patient was the development of thyroid PGL instead of PCC.

Head and neck PGLs in the clinical context of MEN 2 syndrome are extremely rare, with only three cases reported in the literature, concerning a glomus tympanicum [17], a carotid body PGL [18], and a glomus jugulare [12]. Thyroid PGL associated with MEN 2B syndrome is unique. Furthermore, the prolonged survival of 19 years is remarkable, despite unfavorable prognostic factors, such as advanced stage with distant lymph node metastases at diagnosis and early development of disseminated metastatic disease with very high serum calcitonin and CEA levels [19]. Therapy with vandetanib achieved stable disease with manageable toxicity during the last 10 years. A recent study has shown that the majority of children and adolescents with advanced MEN 2B associated with MTC maintained good clinical response to vandetanib [20].

## Conclusions

The present case is the first report of an intrathyroid PGL coexisting with MTC presenting as a manifestation of MEN 2B syndrome. Thyroid PGL is an entity of clinical significance that should be accurately diagnosed not only for the appropriate treatment but also for its strong association with genetic syndromes. Molecular studies are required in patients and family members to further elucidate the possible genetic background of such unusual cases. Tyrosine kinase inhibitors have changed the treatment landscape in advanced, metastatic MTC, as was true in our patient, who showed good clinical response with exceptional prolonged survival.
